# The therapeutic applications of mesenchymal stromal cells from human perinatal tissues in autoimmune diseases

**DOI:** 10.1186/s13287-021-02158-3

**Published:** 2021-02-04

**Authors:** Chao Yang, Mingjun Wu, Min You, Yu Chen, Maowen Luo, Qiang Chen

**Affiliations:** 1 Stem Cells and Regenerative Medicine Research Center, Sichuan Stem Cell Bank/Sichuan Neo-life Stem Cell Biotech Inc., 15 Jinquan Road, Chengdu, 610036 China; 2 Center for Stem Cell Research & Application, Institute of Blood Transfusion, Chinese Academy of Medical Sciences and Peking Union Medical College, Chengdu, China

**Keywords:** Perinatal tissues, Mesenchymal stromal cell, Umbilical cord, Placental membranes, Amniotic fluid, Immune diseases

## Abstract

The autoimmune diseases are characterized by overactivation of immune cells, chronic inflammation, and immune response to self-antigens, leading to the damage and dysfunction of multiple organs. Patients still do not receive desired clinical outcomes while suffer from various adverse effects imparted by current therapies. The therapeutic strategies based on mesenchymal stromal cell (MSC) transplantation have become the promising approach for the treatment of autoimmune diseases due to the immunomodulation property of MSCs. MSCs derived from perinatal tissues are collectively known as perinatal MSCs (PMSCs), which can be obtained via painless procedures from donors with lower risk of being contaminated by viruses than those MSCs from adult tissue sources. Therefore, PMSCs may be the ideal cell source for the treatment of autoimmune diseases. This article summarizes recent progress and possible mechanisms of PMSCs in treating autoimmune diseases in animal experiments and clinical studies. This review also presents existing challenges and proposes solutions, which may provide new hints on PMSC transplantation as a therapeutic strategy for the treatment of autoimmune diseases.

## Introduction

Mesenchymal stromal cells (MSCs), a kind of cells with self-renewal and multiple differentiation characteristics, have a broad application prospect in the field of regenerative medicine [1]. A series of MSCs have been isolated from different tissue sources, including the bone marrow, adipose, umbilical cord, umbilical cord blood, placenta, amniotic fluid, hair follicle, and dental pulp tissue [2–7] Among them, the umbilical cord, umbilical cord blood, placenta, and amniotic fluid are the sources that can be collected at the perinatal period, and the MSCs from these tissues are collectively known as perinatal MSCs (PMSCs). Bone marrow-derived MSCs (BMSCs) are the classical adult MSCs and have become the reference to define the biological characteristics of MSCs from various other sources. Previous studies have demonstrated that MSCs from umbilical cord tissues show less capacity in proliferation and differentiation toward adipocyte and osteocyte lineages, but MSCs are less immunogenic with higher immunosuppression activity compared with BMSCs [8, 9]. Furthermore, PMSCs can be obtained via painless procedures from donors with lower risk of being contaminated by viruses than MSCs from other adult tissue sources [9, 10]. Therefore, PMSCs are considered as one of the candidate cell sources for clinical applications.

The developing embryo is connected to the placenta by the umbilical cord and immersed in amnion fluid. The amnion fluid is in a sac comprised of the amnion, chorion, etc. [11]. Nowadays, several cell types including MSCs and epithelial cells can be isolated from amnion fluid, and amniotic fluid-derived stem cells (AFSCs) have been considered as a potential candidate for cell transplantation and therapy [12]. Although amniotic fluid-derived MSCs (AFMSCs) may have advantages concerning harvesting method and propagation rate [4], MSCs from the umbilical cord (UCMSCs) and placenta (PDMSCs) are used more widely than AFSCs in reviewed research studies and clinical trials using PMSCs. The umbilical cord blood contains limited MSCs (UCBMSCs). Additionally, cryopreservation of the umbilical cord blood in banks do not need to isolate the MNCs using Ficoll-Hypaque-Plus solution; it only requires removing red cells and excessive nucleated cell-poor plasma through hetastarch and centrifugation. Isolation of UCBMSCs from the umbilical cord blood needs to isolate the MNCs using Ficoll-Hypaque-Plus solution and seed into culture plates with medium formulated for MSCs to select for UCBMSCs. This procedure is not compatible with that for hematopoietic stem cells. It may be possible to isolate hematopoietic stem cells with CD34+ magnetic beads prior to seeding into culture plates for UCBMSCs, but it is not the current standard procedure in umbilical cord blood banks [13–15]. Therefore, isolating UCBMSCs may affect the storage of hematopoietic stem cells in umbilical cord blood banks, which restricts the application of UCBMSCs. In short, the umbilical cord, especially Wharton’s jelly compartment, and placenta have become the important sources to isolate MSCs for therapy.

**Fig. 1:**
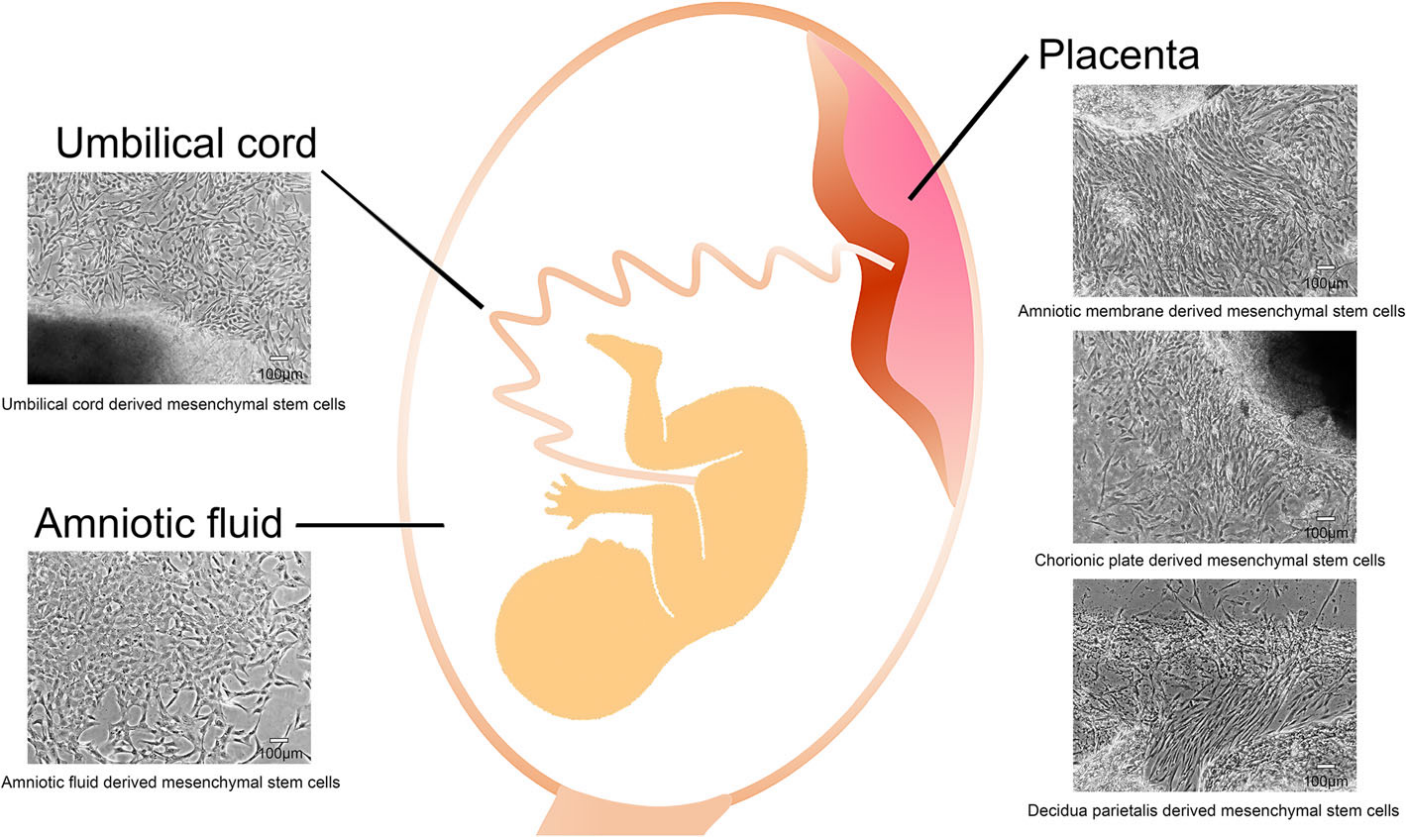
The main human perinatal tissues to obtain mesenchymal stromal cells (MSCs). Umbilical cord- and placenta-derived MSCs can be acquired via tissue explant adherent cultivation method, while amniotic fluid-derived MSCs can be collected by concentration and cultivation. The MSCs from these tissues present the classic spindle shape and adhere to plastic. Additionally, MSCs can be isolated from different membranes of the placenta or different compartments of the umbilical cord

Although UCMSCs are derived from the umbilical cord, a previous study has shown that MSCs from different compartments, including umbilical amnion (AMMSCs), subamnion (SAMSCs), perivascular (PVMSCs), Wharton’s jelly (WJMSCs), and mixed umbilical cord (MCMSCs), have diverse biological properties. WJMSCs show less non-stem cell contaminants, but more stemness characteristics and differentiation potential than PVMSCs, SAMSCs, AMMSCs, and MCMSCs [16]. Meanwhile, our previous study showed that MSCs from the umbilical cord and different membranes of the placenta also exhibited dissimilar biological characteristics. For instance, MSCs derived from decidua parietalis (DPMSCs) are of maternal origin, but MSCs from the umbilical cord, amniotic membrane (AMMSCs), and chorionic plate (CPMSCs) are of fetal origin. Additionally, AMMSCs exhibit better capacity in the secretion of prostaglandin E2 (PGE2) and transforming growth factor β1 (TGF-β1) related to immunomodulation, whereas CPMSCs secrete higher levels of hepatocyte growth factor (HGF) and vascular cell adhesion molecule-1 (VCAM-1), and DPMSCs release higher levels of vascular endothelial growth factor (VEGF) and angiopoietin-1 (ANG-1) [3]. Differences in secretion potentially relate to differences in biological activity, ultimately providing choice of suitable MSCs from the various tissue sources or compartments related to clinical requirements and applications. Figure 1 presents the main human perinatal tissues from which PMSCs can be obtained. The similarities and differences of biological characteristics in MSCs from different perinatal tissues are summarized in Table 1.

**Table 1:** The similarities and differences of biological characteristics in MSCs from different perinatal tissues

Perinatal tissues		Similarities	Differences
Umbilical cord and compartments [16]	Wharton’s jelly (WJMSCs)	1. Adherence to plastic; 2. Positive for SSEA-3, SSEA-4, Tra-1-60, and Tra-1-81; 3. Adipogenic potential and levels of telomerase activity at passage 1.	1. WJMSCs show significantly greater numbers in primary culture compared to other MSCs; 2. WJMSCs show significantly greater proliferation rates at passages 3, 5, and 10 compared to other MSCs; 3. The telomerase levels for WJMSCs were significantly greater than other MSCs at passage 10; 4. WJMSCs express significantly greater for CD29, CD44, CD73, HLA-ABC, CD24, CD108, OCT1, OCT4A, OCT4B, NANOG, and SOX2 compared to other MSCs; 5. WJMSCs and PVMSCs express significantly greater for CD146 and CD271 compared to other MSCs; 6. WJMSCs and PVMSCs express significantly less for fibroblast-related genes FAP, FSP, CD40, CD49d, and CD140b compared to other MSCs; 7. WJMSCs exhibit greater osteogenic and chondrogenic differentiation potential compared to other MSCs.
	Amnion (AMSCs)		
	Subamnion (SAMSCs)		
	Perivascular (PVMSCs)		
	Mixed cord (UCMSCs)		
Umbilical cord and membranes of the placenta [3]	Umbilical cord (UCMSCs)	1. Adherence to plastic; 2. Positive for CD73, CD90, CD105, SOX2, and SSEA4 3. Negative for CD45, HLA-DR, CD34, and CD14; 4. Adipogenic, osteogenic, and chondrogenic differentiation potential.	1. CPMSCs express greater for CD106 compared to other MSCs; 2. AMMSCs, CPMSCs, and UCMSCs were of fetal origin, and DPMSCs were of maternal origin; 3. UCMSCs show shorter population doubling time than other MSCs; 4. AMMSCs exhibit better capacity in the secretion of PGE2 and TGF-β1; 5. CPMSCs secrete higher level of HGF and VCAM-1; 6. DPMSCs release higher levels of VEGF and ANG-1.
	Amniotic membrane (AMMSCs)		
	Chorionic plate (CPMSCs)		
	Decidua parietalis (DPMSCs)		
Umbilical cord and amnion fluid [4]	Wharton’s jelly (WJMSCs)	1. Adherence to plastic; 2. Growth characteristics at passage 5; 3. Positive for the expression of CD29, CD105, HLA-ABC, and OCT4; 4. Negative for CD34 and HLA-DR; 5. Myocardial-like cells differentiation capacity in vitro.	1. AFMSCs show greater growth characteristics at passage 10 than WJMSCs.
	Amnion fluid (AFMSCs)		
Placenta and umbilical cord blood [7]	Placenta (PMSCs)	1. Adherence to plastic; 2. Positive for the expression of CD44, CD73, CD90, CD105, and KLF4; 3. Negative for CD14, CD34, and CD45; 4. Growth characteristics; 5. Adipogenic, osteogenic, and chondrogenic differentiation potential; 6. Immunomodulatory effects.	1. PMSCs are positive for the expression of NANOG; 2. UCBMSCs are positive for expression of MYC.
	Umbilical cord blood (UCBMSCs)		

As a matter of fact, PMSCs are not just used as seed cells for tissue engineering; they exhibit extraordinary capacity in immunomodulation through the secretion of high levels of paracrine cytokines and direct contact mediated by cell adhesion molecules [17–21]. Therefore, MSCs have been used for many immune diseases such as graft-versus-host disease after hematopoietic stem cell transplantation and cytokine storm in critical patients with coronavirus disease 2019 (COVID-19) [22, 23]. Deservedly, MSCs can be also used for the autoimmune diseases, including systemic lupus erythematosus (SLE), lupus nephritis (LN), rheumatoid arthritis (RA), multiple sclerosis (MS), and so forth [24–29]. To date, lots of drugs including antibodies for B lymphocyte, immunosuppressive agents, corticosteroids, and non-steroidal drugs have been used for the treatment of autoimmune diseases [30]. However, many adverse effects such as serious infection, malignant tumorigenesis, and femoral head necrosis limit the clinical application of these drugs [31, 32]. Thus, PMSC transplantation has been considered as a promising strategy for the treatment of various autoimmune diseases. This review mainly summarizes progress and possible mechanisms of MSCs from perinatal tissues to treat autoimmune diseases in animal experiments and clinical studies for the past 15 years and discusses the existing challenges and their possible solutions. The key words for search at “https://pubmed.ncbi.nlm.nih.gov/” are “autoimmune diseases,” “perinatal tissues,” “stem cell,” “mesenchymal stem cell,” “mesenchymal stromal cell,” “MSCs,” “umbilical cord,” “umbilical cord blood,” “placenta,” “decidua parietalis,” “amniotic membrane,” “chorionic plate,” “amniotic fluid,” “systemic lupus erythematosus,” “lupus nephritis,” “rheumatoid arthritis,” “multiple sclerosis,” “experimental autoimmune encephalomyelitis,” “psoriasis,” “primary Sjögren’s syndrome,” “type 1 diabetes,” and their combinations. The references were published from 2005 to 2020, mainly from 2010 to 2020. The referred articles include original studies, clinical trials, reviews, and meta-analysis. Initially, 151 articles were evaluated for inclusion, and 115 articles were included in the final version.

## Pre-clinical applications in autoimmune diseases

### Systemic lupus erythematosus

As a typical autoimmune disease, SLE is characterized by overactivation of B and T lymphocytes, accompanied by secretion of large amounts of inflammatory cytokines and autoantibody, resulting in the damage and dysfunction of multiple organs [33].

Previous studies reported that olfactory 1/early B cell factor–associated zinc-finger protein (OAZ) was highly expressed in SLE patients, which impairs the function of MSCs to inhibit B cell proliferation and terminal differentiation, leading to increased levels of IgG, IgM, and anti-nuclear antibodies. Silencing of OAZ expression restored the immunosuppressive effect of MSCs on B cells. Therefore, it was speculated that transplantation of normal MSCs might suppress the B cell activity and reduce autoantibody production in SLE patients to alleviate the related symptom [34, 35].

T lymphocytes (CD3+) mainly contain two subsets, CD4+ T helper (Th) cells and CD8+ cytotoxic T (Tc) cells. Previous studies have demonstrated that T cells from SLE patients have defective mitochondria with mitochondrial hyperpolarization, ATP depletion, and increased apoptosis compare to those from healthy donors [36, 37]. Chen et al. have also confirmed that SLE patients have excessive mitochondria within T cells, and these T cells also have overactivated autophagy and are prone to apoptosis after stimulation with anti-CD3/C28 antibodies. UCMSCs transfer mitochondria to activate T cells after co-culture for 12 h, which inhibits respiratory mitochondrial accumulation and autophagy activation in T cells, leading to the rescue of T cells from apoptosis [38]. Another study has demonstrated that UCMSCs can induce CD4+ T cell apoptosis in vitro and in vivo, which also reduces the ratio of Th1 to Th2 cells to further suppress the humoral inflammatory response [39], and PGE2 secreted by UCMSCs participates in this apoptosis-promoting process [40]. These results indicate that UCMSCs inhibit autophagy but promote CD4+ T cell apoptosis to achieve the therapeutic effects. Besides CD4+ T cells, CD8+ T cells also play a crucial role both in the initiation and promotion of autoantigen-specific humoral immunity in SLE patients. A previous study demonstrated that the levels of indoleamine 2, 3-dioxygenase (IDO) secreted by allogeneic UCMSCs were enhanced by high levels of interferon-γ (IFN-γ), which was mainly released by CD8+ T cells of SLE patients. The proliferation of T cell was inhibited in patients merely by this large amount of IDO. Therefore, CD8+ T cell/IFN-γ/IDO axis is critical for mediating the therapeutic effects of allogeneic UCMSCs in SLE patients [41]. However, it is interesting that autologous BMSCs from the patients are defective in IDO secretion in response to IFN-γ, suggesting autologous BMSCs might not be a suitable therapeutic cell source for SLE [41]. Later, a clinical study proved that higher baseline levels of IFN-γ might predict a good response to UCMSCs in SLE patients, indicating baseline levels of IFN-γ could be one of the biomarkers to establish the clinical inclusion/exclusion criteria for UCMSC application in SLE patients [42].

Regulatory T (Treg) cells are an immunosuppressive subset of CD4+ T cells and responsible for maintaining immune homeostasis and self-tolerance in healthy individuals [43, 44]. Previous study has demonstrated the reduction and dysfunction of Treg cells in SLE patients [45]. Meanwhile, histocompatibility locus antigen-G (HLA-G), also named as human leukocyte antigen-G, has been proved to be involved in the immunosuppressive properties of MSCs [46]. Soluble forms of HLA-G (sHLA-G) have been demonstrated to contribute to the proliferation of Treg cells in vitro. Later another study found that the levels of sHLA-G in serum increased significantly along with the upregulation of Treg cells after allogenic UCMSC transplantation in SLE patients, indicating that UCMSCs may upregulate Treg cells via sHLA-G [47]. Darlan et al. found that TGF-β1 released by MSC also promoted the generation of inducible Treg (iTreg) cells in the peripheral blood mononuclear cells (PBMCs) of human SLE patients [48].

In addition to Treg cells, T helper 17 (Th17) cells also play an important role in the pathogenesis of SLE [49]. This process involves the imbalance of Treg and Th17 cells in particular. It is interesting that another study showed that IL-17 production from CD4+ T cells, which was purified from PBMCs, in both healthy donor and SLE patients were upregulated by PGE2 and IL-1β secreted by UCMSCs [50]. These results suggested that the other subpopulations besides CD4+ T cells might suppress the activity of Th17 cells and the secretion of IL-17. Furthermore, UCMSCs also significantly upregulate peripheral tolerogenic blood CD1c+ dendritic cells (DCs) and promote CD206 expression and phagocytic activity of macrophages to alleviate SLE [24, 51].

### Lupus nephritis

LN is a potentially destructive outcome and major risk factor for morbidity and mortality in SLE [52]. LN is characterized by conventional clinical parameters, including proteinuria, high level of anti-dsDNA, and anti-nuclear antibodies [53]. The overactivation of various immune cells and overproduction of related proteins or antibodies play critical roles in the pathogenesis of LN. UCMSCs and PDMSCs have been reported to exhibit the therapeutic effect to decrease the extent of renal injury and improve the clinical parameters.

Overproduction of monocyte chemoattractant protein-1 (MCP-1) and high-mobility group box chromosomal protein 1 (HMGB-1) has been found to act as the important factors for LN [54, 55]. Gu et al. reported that UCMSC transplantation alleviated the severity of proteinuria, downregulated the levels of serum creatinine and anti-dsDNA antibody, and inhibited the expression of MCP-1 and HMGB-1. Additionally, UCMSCs could be found in the kidney of the MRL/lpr mice for at least 11 weeks [56]. Podocyte injury, which is typically involved in nephropathy with proteinuria, contributes to the pathogenic development of LN. UCMSCs not only reduce immunoglobulin G (IgG) and C3 deposition in glomeruli and decrease anti-dsDNA antibody levels, but also induce the macrophages exhibiting an anti-inflammatory phenotype to prevent podocyte injury [57]. In addition to UCMSCs, PDMSCs can also reduce the severity of proteinuria and the production of anti-dsDNA antibodies in addition to ameliorating renal pathological changes by inhibiting the expression of inflammatory cytokines, including nuclear factor kappa B (NF-κB), tumor necrosis factor α (TNF-α), and so on [58]. Furthermore, besides untreated MSCs used for LN, hydroxychloroquine-pretreated UCMSCs have also been researched to obtain better therapeutic effect than that of untreated UCMSCs, suggesting appropriate pretreatment of MSCs before transplantation can promote better clinical outcomes [59].

### Rheumatoid arthritis

RA is a chronic autoimmune joint disease and characterized by invasive synovium inflammation, leading to cartilage and bone destruction and even disability. Although some patients can now benefit from antirheumatic drugs, many still do not receive desired clinical outcomes from current therapies. Therefore, either new therapies need to be developed, or current ones improved urgently [60]. Many studies have reported that UCMSC and UCBMSC transplantation could be potential therapeutic strategies for the treatment of RA.

UCMSCs benefit RA patients through regulating the ratio/function of immune cells and decreasing inflammatory cytokines in joints and serum. Previous studies demonstrated that UCMSCs inhibit the proliferation and IL-6 secretion of fibroblast-like synoviocytes (FLSs) but promote Treg cell expansion in RA patients by secreting IL-10, IDO, and TGF-β1. Further animal experiments determined that UCMSCs downregulated the levels of TNFα, IL-6, and MCP-1, and upregulated the levels of IL-10, leading to the relief of the severity of collagen-induced arthritis (CIA) [61, 62]. In addition, cadherin-11 (CDH11) is a type II cadherin predominantly expressed by FLSs and is involved in the pathogenesis of RA. UCMSCs could inhibit the expression of CDH11 via IL-10 to prevent cartilage erosion [63]. Abnormal activation and differentiation of T lymphocytes is one of the crucial inducements of inflammation, cartilage, and bone destruction in RA. UCMSCs inhibit the expansion and promote the apoptosis of T lymphocytes and decrease the ratio of Th17/Treg cells in the spleen and joints of CIA rats. Moreover, UCMSCs also decrease the levels of pro-inflammatory IL-17 but increase the levels of anti-inflammatory TGF-β in serum to ameliorate symptoms [64]. It is interesting that chemical molecules, such as quercetin, may upregulate the immunomodulatory effect of UCMSCs and improve the outcomes by increasing the functional molecules, including nitric oxide, IDO, and IL-6 via activation Toll-like receptor-3 signaling, suggesting pretreated MSCs may be a potential therapeutic for the treatment of RA in the clinic [65].

Similar to UCMSCs, UCBMSCs have also been reported to significantly downregulate pro-inflammatory cytokines, including TNF-α, IL-1, and IFN-γ, but upregulate IL-10. Histologic analysis reveals that UCBMSCs reduce the infiltration of immune cells and hypertrophy of the synovial tissue, ultimately decrease the severity of arthritis induced by Freund’s complete adjuvant [66]. Furthermore, macrophages are responsible for the pathogenesis of RA via producing core inflammatory cytokines [67]. Shin et al. found that UCBMSCs could polarize naive macrophages toward an M2 phenotype via regulating the production of TNF-α and IL-1β [68].

### Multiple sclerosis and experimental autoimmune encephalomyelitis

MS is an autoimmune disorder in the central nervous system and characterized by chronic inflammation and multifocal demyelination [69]. In recent decades, the clinical strategy for the treatment of MS remain relatively disappointing and face many challenges [70]. MSC transplantation is a promising therapeutic strategy for future treatment of MS, and experimental autoimmune encephalomyelitis (EAE) is currently the appropriate animal model for MS research.

UCMSC transplantation attenuated the behavioral-deficit scores through ameliorating demyelination and axonal damage, promoting remyelination in the spinal cord of EAE mice via trophic support properties, and modulation of immune cell function [71, 72]. Moreover, UCMSCs exhibit immunoregulation effect to modulate the ratios of Th1/Th2 cells and Th17/Treg cells in the spleen, leading to increased IL-4 and IL-10 but decreased IL-1 and IL-6 in the spinal cord [72]. Although most of the UCMSCs are cleared away, a fraction of cells can survive up to 3 weeks in the lung and spleen [71]. A similar therapeutic effect is also presented in the primate model of EAE; UCMSCs ameliorate clinical signs of EAE and reduce inflammation and demyelination in the central nervous system via promoting anti-inflammatory immune cells and cytokines while inhibiting pro-inflammatory immune cells and cytokines, and suppressing astrocyte activation in cynomolgus monkeys of EAE [73].

Gene modification and reagent stimulation are used to improve the therapeutic effect of UCMSCs in EAE models. Sphingosine kinase 1 (SPK1) catalyzes sphingosine phosphorylation process to produce sphingosine 1-phosphate, which plays a critical role in cell proliferation, migration, and immune regulation [74]. Wang et al. reported that SPK1-transfected UCMSCs presented better therapeutic activity than wildtype UCMSCs in the EAE model, including reducing the severity of neurological deficits, inhibiting myelin oligodendrocyte glycoprotein (MOG)-specific T cells and NK cells, and promoting Treg cells [75]. Tetramethylpyrazine (TMP) as an alkaloid monomer extracted from traditional Chinese herbs can facilitate the proliferation of neural stem cells and have anti-inflammatory and anti-apoptotic properties. Zhang et al. demonstrated that TMP not only increased cell viability but also reduced intracellular reactive oxygen species (ROS) production via nuclear factor-erythroid 2-related factor-2 (Nrf2)/heme oxygenase 1 (HO-1) signaling. Further experiments determined that TMP-treated UCMSCs also exhibited better effects on the attenuation of inflammation, demyelination, and blood–brain barrier disruption than those of untreated UCMSCs [76]. However, not all of the modification and reagent stimulation of MSCs are beneficial to the amelioration of EAE. Although IFN-γ, IL-1β, and TNF-α pretreatment (called licensing) increase the expression of immune-modulatory molecules and enhance the immunosuppressive potential of UCMSCs, in vitro licensed UCMSCs do not ameliorate EAE. This above study also confirmed that licensing alters the surface marker profile of UCMSCs toward a more immunogenic phenotype, suggesting that a fast rejection is earlier than the immunomodulation effect of licensed UCMSCs, which may cause the failure of treatment [71].

PDMSCs also play a positive role in the treatment of EAE. Firstly, PDMSCs can decrease the levels of IL-17 and increase the production of IL-4 and IL-10 to reduce inflammation partly through tumor necrosis factor α stimulated gene-6 (TSG-6) activation [77, 78]. Secondly, PDMSCs promote the expression of neurotrophic factors such as brain-derived neurotrophic factor, nerve growth factor, and neurotrophin 3 in the brains of EAE rats [79]. Thirdly, PDMSCs can migrate into inflamed tissues and express neural–glial lineage markers while maintaining the anti-inflammatory, axon-preserving, and demyelination-ameliorating properties [80]. Of particular attention is that the route of administration of MSCs influences the therapeutic outcome of EAE. Previous studies demonstrated an improvement in disease symptoms following intravenous, intracerebral, and intraperitoneal injection of MSCs in the EAE mice [71, 73, 77, 78]. Later, Shapira et al. examined the systemic delivery of PDMSCs via intramuscular implantation due to the low risk of pulmonary obstruction through this route. However, intramuscular PDMSC implantation exhibited only marginal effect in inhibiting neuroinflammation and no effect in treating established disease [81]. Additionally, the previous study also revealed that functional peptides and protein co-transplantation could improve the therapeutic effect; for instance, synthetic C16 peptide and ANG-1 increased the engraftment efficacy of PDMSC in the CNS and promoted expression of the neurotropic factors and differentiation of the transplanted PDMSCs [82].

### Psoriasis

Psoriasis is a chronic and systemic disorder with abnormal keratinocyte proliferation and immune cell infiltration in the dermis and epidermis which seriously affect the patients’ quality of life [83]. The previous study investigated the possible therapeutic mechanisms and demonstrated that UCMSCs ameliorated the skin inflammation in an imiquimod-induced psoriasis model, and attenuated the production of type I IFN by plasmacytoid DCs [84]. Not only that, another study also found that UCBMSCs treated DCs repressed the activation and differentiation of CD4+ T cells which are important for the pathogenesis of psoriasis [85], suggesting that these MSCs not only repress DC function but also alter the immune properties. Furthermore, pro-inflammatory cytokines and chemokines, including IL-6, IL-17, TNF-α, chemokine (C-C motif) ligand (CCL) 17, CCL20, and CCL27 were downregulated after treatment [84, 85].

Besides UCMSCs and UCBMSCs, AMSCs can also attenuate epidermal acanthosis and neutrophil infiltration of the epidermis in imiquimod-induced psoriasis mice. Moreover, AMSCs suppress the expression of CXC chemokine ligand (CXCL) 1 and downregulate IL-17A and IL-22 production from γδ-low T cells. It is interesting that AMSC conditioned medium also inhibits the IL-8 secretion by human epidermal keratinocytes induced by IFN-γ and TNF-α [86].

### Primary Sjögren’s syndrome

Primary Sjögren’s syndrome is a systemic autoimmune disease characterized by aberrant activation of immune responses and lymphocytic infiltration of the salivary and lacrimal glands, leading to xerostomia and xerophthalmia [87, 88]. Recent studies have reported that UCMSCs alleviate the related symptoms via mediating the functions of T17 cells, Treg cells, T follicular helper (Tfh) cells, and macrophages.

The imbalance of Treg/Th17 cells has been proven to be involved in the development of autoimmune diseases [89]. Alunno et al. found that IFN-γ-pretreated microencapsulated-UCMSCs suppress T cell proliferation and restore the Treg/Th17 ratio in PBMCs from primary Sjögren’s syndrome patients [90]. And not only that, UCMSCs also downregulate the production of pro-inflammatory cytokines including IFN-γ, IL-6, and TNFα but upregulate the production of IL-10, leading to the reduction of inflammatory infiltrates and the increase of salivary flow [91]. Tfh cells are a specialized CD4+ T cell subset, which are essential for B cell maturation [92]. The frequency of circulating Tfh cells correlates with serum autoantibody levels in patients [93]. UCMSCs suppress the differentiation and proliferation of Tfh cells via the secretion of IDO, whereas the production of IDO by UCMSCs is also promoted by naive CD4+ T cells under Tfh cell-polarizing conditions with cell-to-cell contact but not in a trans-well system [88]. Macrophages are important cells for immune system, and the accumulation of macrophages in lacrimal glands and salivary glands has been found in primary Sjögren’s syndrome patients [94]. Lu et al. demonstrated that UCMSCs activated AKT pathway in macrophages and skewed macrophages into an M2 phenotype, resulting in the downregulation of pro-inflammatory M1 macrophage and the upregulation of anti-inflammatory M2 macrophage, and alleviation of chronic inflammation in lacrimal glands [95].

### Type 1 diabetes

Type 1 diabetes (T1D) is a chronic autoimmune disease and characterized by pancreatic β cell damage, insulin deficiency, and hyperglycemia [96]. Current therapeutic strategies focus on intensifying insulin and preserving β cell mass, but different to maintain normal glycemic levels and transient efficacy, respectively [28]. Although T1D remains a huge therapeutic challenge because of the wide gaps in our understanding of this disease, T cell-mediated destruction of pancreatic β cells is one mode of the pathogenesis. MSC transplantation has been reported to treat T1D by the immunomodulatory property of MSCs.

Although human AFSCs have not been used in T1D clinical trials, non-genetically engineered AFSCs have been reported to protect β cell from damage and promote β cell regeneration in streptozotocin-induced diabetic mice via activation of the insulin receptor/PI3K/Akt signaling pathway and upregulation of vascular endothelial growth factor-A (VEGF-A) expression [97].

MSCs can differentiate into insulin-producing cells and have given rise to a new approach to replace the damaged pancreatic β cells. Previous studies demonstrated that human AMMSCs could differentiate into insulin-producing cells after cultivation in induction medium or knocking down neuronal restrictive silencing factor (NRSF) and Sonic hedgehog (SHH) via PEI@Fe3O4 nanoparticles. The insulin-producing cells can release insulin in a glucose-responsive manner and normalize hyperglycemia for a long period without immunosuppressive agents [98, 99]. In addition to AMSCs, human AFSCs can also be induced to differentiate into insulin-producing cells; the induced AFSCs can secret insulin in response to glucose stimulation just like intrinsic pancreatic β cells [100].

## Clinical trials in autoimmune diseases

### Systemic lupus erythematosus

Safety is the primary consideration in clinical trial of allogeneic UCMSC transplantation for SLE. Many long-term follow-up clinical studies provide evidences that the incidences of adverse events, concerning renal function, liver function, electrocardiogram, infections, malignancies, and so forth, are acceptable, suggesting that allogeneic UCMSC transplantation exhibits a good safety profile in SLE patients [101–103]. A clinical study for 12 months demonstrated that after UCMSC transplantation in active SLE patients, the percentage of peripheral Treg cells increased in the initial 3 months, while the proportion of Th17 cells decreased in the last 9 months. This study also showed that UCMSCs could upregulate the proportion of Treg cells and downregulate the percentage of Th17 cells, but could not influence the production of interleukin (IL)-17A [104]. In addition, UCMSC transplantation elicits a satisfactory clinical response in SLE patients, but repeated infusion for every 6 months is necessary to avoid disease relapse for some patients [105].

### Lupus nephritis

Clinical study showed that multiple assessment indicators, including renal activity, renal function, glomerular filtration rate, and total disease activity, were ameliorated after a single intravenous infusion of UCMSCs (1 × 10^6^ cells per kilogram), and this amelioration lasted for 12 months without transplantation-related adverse events, suggesting that allogeneic UCMSC transplantation is safe and efficacious for active and refractory LN patients [106]. However, another clinical study demonstrated that compared with the UCMSC transplantation (2 × 10^8^ cells per patient) group, the placebo group also achieved a similar complete remission proportion of patients [25]. Some researchers proposed that the intravenous infusion approach made the UCMSCs contact patient’s immune cells directly, which hampered the immunomodulatory function [107].

### Rheumatoid arthritis

Clinical trials demonstrate that there is no serious adverse effect associated with UCMSC transplantation at the dose of 2~4 × 10^7^ or UCBMSC transplantation at the dose of 2.5~10 × 10^7^ [26, 108, 109]. Additionally, UCMSCs or UCBMSCs decrease the serum levels of TNF-α and IL-6 and increase the percentage of Treg cells in the peripheral blood, according to the results from in vitro and animal RA models [109]. All the clinical trials demonstrate that UCMSC or UCBMSC transplantation induces a significant remission of clinical symptoms of RA, indicating that they are safe and effective approaches for the treatment of RA [26, 108, 109].

### Multiple sclerosis

A phase 1b clinical study of MS demonstrated that PDMSC transplantation at the dose of 1.5~6 × 10^8^ cells were safe and well tolerated in relapsing–remitting multiple sclerosis and secondary progressive multiple sclerosis patients via intravenous infusion [27].

### Psoriasis

Chen et al. reported two clinical cases of patients who had suffered psoriasis vulgaris more than 10 years and then treated by UCMSCs. UCMSC transplantation significantly reduced the severity and development of psoriasis at least for 4 years [110].

### Type 1 diabetes

Hu et al. reported that both the HbA1c and C peptide in TID patients with UCMSC transplantation were significantly better than those in the TID patients treated with saline for 21 months, suggesting that UCMSC transplantation is expected to be an ideal therapeutic strategy for T1D [111]. Another clinical study establishes co-transplantation of allogenic UCMSCs with autologous bone marrow mononuclear cells (BMMNCs) without immunotherapy. C-peptide area under the curve and insulin area under the curve are increased, whereas HbA1c, fasting glycemia, and daily insulin requirements decreased after co-transplantation [28]. These results reveal that transplantation of UCMSC alone or with autologous BMMNCs is safe and promotes the improvement of metabolic measures in T1D patients.

The pre-clinical results and clinical outcomes for each autoimmune disease with respect to PMSC types are summarized in Table 2.

**Table 2:** The pre-clinical results and clinical outcomes for each autoimmune disease with respect to PMSC types

Type of autoimunne diseases	Type of PMSCs	Pre-clinical results	References
SLE	UCMSCs	Inhibiting B cell activity and autophagy activation in T cells; inducing CD4+ T cell apoptosis; upregulating the ratio of Th2/Th1; promoting generation of Treg cells, CD1c+DCs, and CD206+ M2 macrophages	[24, 34, 38, 39, 47, 48, 51]
LN	UCMSCs	Downregulating the levels of proteinuria, serum creatinine, anti-dsDNA antibody, IgG, and C3 deposition; inducing macrophages to M2 phenotype	[56, 57]
	PDMSCs	Reducing the severity of proteinuria, production of anti-dsDNA antibodies; inhibiting the expression of inflammatory cytokines NF-κB, TNF-α, etc.	[58]
RA	UCMSCs	Decreasing the ratio of Th17/Treg cells; inhibiting the expression of CDH11; downregulating the levels of TNFα, IL-6, MCP-1, and IL-17, and upregulating the levels of IL-10 and TGF-β	[62–64]
	UCBMSCs	Downregulating TNF-α, IL-1, and IFN-γ, but upregulating IL-10; reducing the infiltration of immune cells and hypertrophy of the synovial tissue	[66, 68]
EAE	UCMSCs	Ameliorating demyelination, axonal damage, and promoting remyelination in the spinal cord; modulating the ratio of Th1/Th2 cells and Th17/Treg cells in the spleen; increasing IL-4 and IL-10 but decreasing IL-1 and IL-6 in the spinal cord	[71–73]
	PDMSCs	Decreasing infiltrating inflammatory cells, preserving axons, and ameliorating demyelination; decreasing the levels of COX-2, NF-kB, TNF-α, IFN-γ, and IL-2, and increasing production of IL-4 and TGF-β	[77, 78, 80]
Psoriasis	UCMSCs	Ameliorate the skin inflammation; attenuating plasmacytoid DCs to produce type I IFN	[84]
	UCBMSCs	Downregulating IL-6, IL-17, TNF-α, CCL17, CCL20, and CCL27	[85]
	AMSCs	Decreasing both IL-17A and IL-22 production by cutaneous γδ-low T cells	[86]
Primary Sjögren’s syndrome	UCMSCs	Suppressing the T cell proliferation and restoring the Treg/Th17 ratio of PBMCs; downregulating the production of IFN-γ, IL-6, and TNFα but upregulating production of IL-10; suppressing the differentiation and proliferation of Tfh cells; inducing macrophages into an M2 phenotype	[88, 90, 91, 95]
T1D	AFSCs	Preserving and promoting endogenous β cell functionality and proliferation	[97]
Type of autoimunne diseases	Type of PMSCs	Clinical outcomes	References
SLE	UCMSCs	Exhibiting a good safety profile in SLE patients; upregulating the ratio of Treg/Th17; 32.5% patients achieved major clinical response and 27.5% patients achieved partial clinical response during 12 months of follow-up. 12.5% and 16.7% patients experienced disease relapse at 9 months and 12 months	[104, 105]
LN	UCMSCs	Exhibiting a good safety profile in LN patients; ameliorating renal activity, renal function, glomerular filtration rate, and total disease activity	[25, 106, 107]
RA	UCMSCs	Exhibiting a good safety profile in RA patients; decreasing health index and joint function index of 1 year and 3 years after treatment; decreasing the serum levels of TNF-α and IL-6; increasing the percentage of Treg cells in peripheral blood	[26, 109]
	UCBMSCs	Exhibiting a good safety profile in RA patients; reducing levels of IL-1β, IL-6, IL-8, and TNF-α; increasing the percentage of Treg cells in peripheral blood; reducing the mean 28-joint disease activity score at week 4	[108]
MS	PDMSCs	Exhibiting a good safety profile in MS patients; decreasing Expanded Disability Status Scale scores	[27]
Psoriasis	UCMSCs	Reducing the severity and development of psoriasis at least 4 years	[110]
T1D	UCMSCs	Increasing levels of C peptide and decreasing HbA1c, fasting glycemia, and daily insulin requirements	[28, 111]

## Present progress and challenges of the future

The therapeutic strategies based on MSC transplantation have become the promising approaches for the treatment of the diseases without satisfactory clinical outcomes provided by current therapies. As mentioned above, PMSCs have been used and received considerable attention for the treatment of various autoimmune diseases without significant adverse effects. The main mechanisms contain mediating the ratio of inflammatory cells to anti-inflammatory cells, inhibiting the levels of inflammatory factors, and promoting anti-inflammatory factors. However, there are also several key points that should be considered for the clinical application in the future.

Firstly, potency evaluation and screening criteria should be established. For instance, MSCs derived from the same type of tissue source have been used for the same disease; however, the results from one clinical trial showed that the patients developed positive clinical outcomes while those from another clinical trial indicated that MSC transplantation did not exhibit desired therapeutic effects [25, 106]. In such case, the difference of the effects might be caused by the potency of MSCs due to the difference in donors or culture system, including medium components, culture environment, technological standard, etc. Therefore, the challenge is how to establish an evaluation system which can predict the efficacy of MSCs after clinical transplantation. In addition to sterility, safety, activity, uniformity, purity, and stability, the released levels of cytokines (IDO, PGE2, TSG-6, IL-4, IL-6, IL-10, VEGF, HGF, ANG-1, fibroblast growth factor 2, placental growth factor, matrix metalloproteinases, CXCLs, CCLs, etc.) and the expression of surface molecules (CD106, CD54, HLA-G, programmed cell death ligand 1, etc.), which related to immunomodulation and regeneration, should be considered [112–114]. Furthermore, inhibition rate of peripheral blood mononuclear cells quantity and levels of inflammatory factors (TNF-α, IFN-γ, etc.) after co-culture with MSCs in vitro should also be included into the potency evaluation system [48]. Certainly, the animal models of indication should be used to prove the utility of the MSC potency evaluation system. The screening criteria should also contain the inclusion criteria of the donors. The previous study demonstrated that MSCs from the same type of tissue source (umbilical cord) but not the same donor exhibited huge differences in the expression profile of immunomodulatory molecules (IOD-1, TSG-6, TGF-β1, etc.), which is closely related to the immune-modulatory function of MSCs [71]. Thus, the selection of the donors is critical, and the potency evaluation system should also be used to screen the donors.

Secondly, the route of injection and the selection of the dose should be considered seriously. Generally, intravenous injection is a convenient and common route for most of the autoimmune disease [26, 27, 102, 108, 111], but it also comes with the risk of adverse events such as pulmonary embolism [115]. Despite being used for the treatment of the EAE animal model in a previous study [77], intraperitoneal infusion has been rarely used for cell delivery for fear of lowering the efficiency. Furthermore, autoimmune diseases are characterized by increased reactivity of the immune system toward self-antigens, resulting in damage or dysfunction of tissues, but different autoimmune diseases usually affect different organs. For instance, LN is characterized by nephritis, RA is characterized by cartilage and bone destruction, and MS is characterized by central nervous system inflammation and multifocal demyelination. Thus, different autoimmune diseases may need different routes of injection, and local injection is also recommended for some autoimmune diseases, for example, intracerebral injection for MS or EAE [78]. For dose selection of MSCs, the climbing dose needs to be designed, which helps to determine the dose–effect relationship, dose-limiting toxicity, and maximum tolerated dose [108]. Additionally, the dose designs are different in previous clinical studies and generally fall into two categories: one is designed according to body weight (for instance 1 × 10^6^/kg) [102], and the other is constant dose per time point or single dose not based on body weight [26, 109].

Finally, as mentioned above, gene modification, reagent stimulation (such as chemical reagents and factors), and microenvironment alteration (such as hypoxia induction) should be considered to improve the immunomodulation capacities of MSCs for the treatment of autoimmune diseases [59, 75, 76, 90]. Nowadays, MSCs obtained from traditional and routine culture system face the bottleneck of limited potency although an evaluation system can screen the relatively better cell sources. These strategies above may further improve the therapeutic potential of screened MSCs. Generally speaking, gene modification has the advantages of forced and targeted expression, but will also face stronger regulatory rules for clinical trials whereas factor stimulation or hypoxia induction is more likely to be accepted currently. In the future, just like chimeric antigen receptor T cells (CAR-T), genetically engineered MSCs may be a trend for therapeutic application. Meanwhile, it is worth considering that some stimulation and alteration may enhance immune-modulatory function of MSCs, but they also may alter the expression profile of MSCs toward a more immunogenic phenotype or other changes in biological characteristics, resulting in the failure of treatment.

## Conclusion

**Fig. 2:**
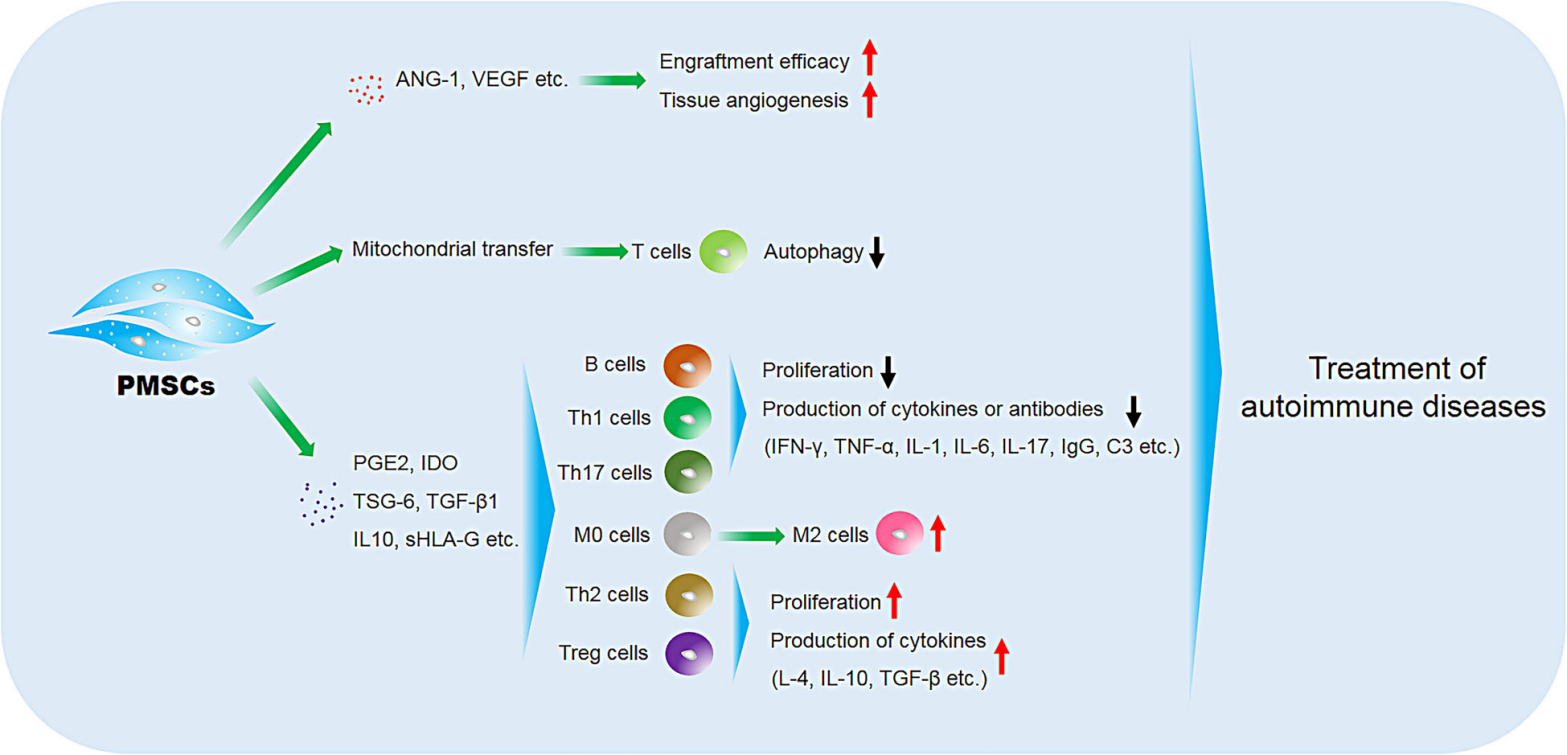
The main mechanisms of PMSCs for the treatment of autoimmune diseases. PMSCs can suppress the function of B and T cells and decrease the proportion of Th1/Th2, Th17/Treg, and M1/M2 via secreting IDO, PGE2, TSG-6, TGF-β1, IL10, sHLA-G, etc. Furthermore, PMSCs can downregulate the levels of inflammatory cytokines as IFN-γ, TNF-α, IL-1, IL-6, IL-17, and the like, but upregulate the levels of IL-4, IL-10, and TGF-β in the serum or target tissues after transplantation. Moreover, PMSCs can secrete various growth factors such as VEGF and ANG-1 to enhance the therapeutic effects

The autoimmune diseases are chronic and systemic disorders and characterized by overactivation of immune cells and chronic inflammation, leading to the damage and dysfunction of multiple organs. However, current therapies for these diseases are based on immunosuppressive agents, corticosteroids, and non-steroidal drugs, which cause many adverse effects. PMSCs exhibit extraordinary capacity in immunomodulation through paracrine route or direct contact, thus can be the candidate for the treatment of autoimmune diseases. As main mechanisms, PMSCs can inhibit the function of B and T cells, decrease the ratios of Th1/Th2, Th17/Treg, and M1/M2 via secreting IDO, PGE2, TSG-6, TGF-β1, IL10, sHLA-G, etc. Additionally, PMSCs can downregulate the levels of inflammatory cytokines such as IFN-γ, TNF-α, IL-1, IL-6, IL-17, and the like, but upregulate the levels of IL-4, IL-10, and TGF-β in the serum or target tissues after transplantation. Moreover, PMSCs can secrete various growth factors to enhance the therapeutic effects—besides immunomodulation; for example, VEGF or ANG-1 enhances the engraftment efficacy and angiogenesis (Fig. 2). The current clinical trials of PMSCs for the treatment of autoimmune diseases indicate that PMSC transplantation exhibits a good safety profile in autoimmune disease patients. Finally, in order to ensure the efficacy, potency evaluation of PMSCs, the route of injection, and dose selection should be thought over. Furthermore, the methods such as gene modification, reagent stimulation, and microenvironment alteration for improving the therapeutic potential should also be considered.

## Data Availability

Not applicable.

## Abbreviations

MSCs: Mesenchymal stromal cells
PMSCs: Perinatal mesenchymal stromal cells
AFSCs: Amniotic fluid-derived stem cells
AFMSCs: Amniotic fluid-derived mesenchymal stromal cells
UCMSCs: Umbilical cord mesenchymal stromal cells
PDMSCs: Placenta-derived mesenchymal stromal cells
UCBMSCs: Umbilical cord blood-derived mesenchymal stromal cells
DPMSCs: Mesenchymal stromal cells derived from decidua parietalis
AMMSCs: Mesenchymal stromal cells derived from amniotic membrane
CPMSCs: Mesenchymal stromal cells derived from chorionic plate
PGE2: Prostaglandin E2
TGF-β1: Transforming growth factor β1
HGF: Hepatocyte growth factor
VCAM-1: Vascular cell adhesion molecule-1
VEGF: Vascular endothelial growth factor
ANG-1: Angiopoietin-1
COVID-19: Coronavirus disease 2019
SLE: Systemic lupus erythematosus
LN: Lupus nephritis
RA: Rheumatoid arthritis
MS: Multiple sclerosis
OAZ: Olfactory 1/early B cell factor–associated zinc-finger protein
Th: T helper cells
Tc: Cytotoxic T cells
IDO: Indoleamine 2, 3-dioxygenase
IFN-γ: Interferon-γ
Treg: Regulatory T cells
HLA-G: Histocompatibility locus antigen-G
sHLA-G: Soluble forms of HLA-G
iTreg: Inducible regulatory T cells
Th17: T helper 17 cells
IL: Interleukin
DCs: Dendritic cells
MCP-1: Monocyte chemoattractant protein-1
HMGB-1: High-mobility group box chromosomal protein 1
IgG: Immunoglobulin G
NF-κB: Nuclear factor kappa B
TNF-α: Tumor necrosis factor α
FLSs: Fibroblast-like synoviocytes
CIA: Collagen-induced arthritis
CDH11: Cadherin-11
EAE: Experimental autoimmune encephalomyelitis
SPK1: Sphingosine kinase 1
MOG: Myelin oligodendrocyte glycoprotein
TMP: Tetramethylpyrazine
ROS: Reactive oxygen species
Nrf2: Nuclear factor-erythroid 2-related factor-2
HO-1: Heme oxygenase 1
TSG-6: Tumor necrosis factor α-stimulated gene-6
CNS: Central nervous system
CCL: Chemokine (C-C motif) ligand
CXCL: CXC chemokine ligand
Tfh: T follicular helper cells
T1D: Type 1 diabetes
BMMNCs: Bone marrow mononuclear cells
VEGF-A: Vascular endothelial growth factor-A
NRSF: Neuronal restrictive silencing factor
SHH: Sonic hedgehog
CAR-T: Chimeric antigen receptor T cells
